# Assessing the Impact of Psychiatric Deinstitutionalization and Substance Use on Patient Outcomes: A Multi-Faceted Analysis

**DOI:** 10.3390/healthcare13141700

**Published:** 2025-07-15

**Authors:** Elena Tanase, Sorina Maria Denisa Laitin, Adrian Cosmin Ilie, Radu Ion, Dan-Alexandru Surducan, Adina Bucur, Felicia Marc, Roxana Folescu, Sorin Ursoniu

**Affiliations:** 1 Doctoral School, Faculty of Medicine, “Victor Babes” University of Medicine and Pharmacy Timisoara, 300041 Timisoara, Romania; tanase.elena@umft.ro; 2 Department of Functional Sciences, Discipline of Public Health, Center for Translational Research and Systems Medicine, “Victor Babes” University of Medicine and Pharmacy Timisoara, 300041 Timisoara, Romania; ilie.adrian@umft.ro (A.C.I.); radu.ion@umft.ro (R.I.); surducan.dan@umft.ro (D.-A.S.); bucur.adina@umft.ro (A.B.); sursoniu@umft.ro (S.U.); 3 Discipline of Epidemiology, Faculty of Medicine, “Victor Babes” University of Medicine and Pharmacy Timisoara, 300041 Timisoara, Romania; laitin.sorina@umft.ro; 4 Department of Medical Sciences, Faculty of Medicine and Pharmacy, University of Oradea, 410073 Oradea, Romania; 5 Discipline of Family Medicine, Faculty of Medicine, “Victor Babes” University of Medicine and Pharmacy Timisoara, 300041 Timisoara, Romania

**Keywords:** mental disorders, deinstitutionalization, substance abuse, quality of life, health services research

## Abstract

**Background and Objectives**: The worldwide shift toward psychiatric deinstitutionalization has aimed to enhance patient autonomy, social integration, and overall quality of life. However, limited studies have examined how concurrent substance use—particularly alcohol, marijuana, and inhalable drugs—affects clinical outcomes in these populations. This study aimed to evaluate psychiatric patients with varying degrees of institutionalization and investigate whether substance use complicates or exacerbates treatment outcomes. We hypothesized that individuals using substances would demonstrate worse psychosocial functioning, higher healthcare costs, and increased readmission rates. **Methods**: We performed a cross-sectional study of 95 participants recruited from long-term care facilities. Participants completed the SF-36 survey validated in Romanian. Financial data were collected to gauge direct and indirect healthcare expenditures. **Results**: Results indicated that 34.7% of participants reported alcohol use, 12.6% used marijuana, and 9.5% used inhalable substances. Substance-using patients experienced higher mean hospitalization costs of approximately USD 3251.8, compared to non-users (USD 2743.6, *p* = 0.032). Quality-of-life scores were significantly lower among substance users (mean SF-36 score 58.4 vs. 66.7, *p* = 0.027). Rates of relapse and readmission were also notably higher in the substance-using cohort (42.1%) relative to non-users (29.8%, *p* = 0.041). **Conclusions**: To our knowledge, this is the first Romanian study—and one of only a handful in Europe—to quantify how specific substance-use profiles simultaneously alter quality of life and direct healthcare costs in a deinstitutionalized psychiatric population. Our findings highlight the need for integrated interventions targeting both mental health and substance abuse.

## 1. Introduction

Over the past few decades, the trend toward deinstitutionalizing psychiatric care has transformed mental-health services worldwide [1,2]. This shift seeks to reduce the stigma of long-term hospitalization while promoting community-based treatment. Many regions struggle with a lack of specialized staff, limited funding, and fragmented services [3,4], leading to questions about the true benefits of deinstitutionalization for diverse patient populations [5].

While psychiatric disorders alone present numerous challenges, the co-occurrence of substance use—particularly involving alcohol, marijuana, and inhalable drugs—can further complicate clinical management [6]. Substance use may exacerbate symptoms, precipitate relapse, and increase the frequency of psychiatric crises [7]. Individuals with dual diagnoses often find themselves in a cycle of repeated hospital admissions, incomplete recovery, and strained social support networks, thereby diminishing the promise of community reintegration [8].

Deinstitutionalization offers ethical and social benefits, yet it remains hampered by fragmented community services, staff shortages, and inconsistent funding. Without reliable follow-up and integration with addiction services, the model’s promised gains can quickly erode [9,10,11,12].

The recent literature emphasizes that mental-health outcomes are influenced not only by clinical treatment but also by socioeconomic factors, patient engagement, and co-occurring behaviors such as substance abuse [13]. In some regions, specialized programs exist to address these overlapping issues [14,15].

In the Romanian context, evolving mental-health policies have aimed to improve community-based services, yet the integration of substance-abuse treatment into psychiatric care remains inconsistent [16]. Many patients traverse multiple care settings, from psychiatric units to social services and back, without a cohesive care plan [17]. The outcome can be suboptimal recovery, recurrent crises, and eventual rehospitalization, perpetuating a costly and distressing cycle [18].

Despite abundant global commentary, no Romanian investigation has yet examined how varying levels of institutional care interact with detailed substance-use profiles to predict both economic burden and patient-reported quality of life. The present study addresses this gap by providing the first national evidence set linking these dimensions across inpatient, community, and residential settings. Given these complexities, it is crucial to examine both the promise and the pitfalls of deinstitutionalization, especially among those who use alcohol, marijuana, or inhalable substances [19]. The present study builds on existing frameworks while adding a focused analysis of substance use as a potential moderator of patient outcomes [20]. We aim to clarify how these combined factors influence quality of life, healthcare utilization, and long-term stability, thereby informing future policy and clinical strategies

## 2. Materials and Methods

### 2.1. Study Design and Setting

This cross-sectional, observational study was conducted between February 2023 and February 2025 in the long-term residential facility for individuals with chronic psychiatric conditions. These sites were selected to capture the full spectrum of institutionalization levels—from high-acuity inpatient care to fully community-based living. Ethical approval was obtained from the Institutional Review Board of “Victor Babeș” University (IRB protocol no. UMFT-PSY-2022-08), and all procedures were conducted in accordance with the Declaration of Helsinki and EU Good Clinical Practice guidelines. Written informed consent was secured from each participant prior to any data collection; for those lacking capacity, consent was obtained from legally authorized representatives under Article 167 of Law No. 95/2006 and Order 904/2006, Article 28.

### 2.2. Participant Selection and Inclusion Criteria

The target population comprised adults (18–65 years) with a DSM-5 diagnosis of schizophrenia spectrum disorder, bipolar disorder, major depressive disorder with psychotic features, or schizoaffective disorder. We conducted a priori sample-size calculation using G*Power 3.1 for detecting a medium effect (Cohen’s d = 0.50) in SF-36 total score between substance-using and non-using groups (α = 0.05, power = 0.80), yielding a minimum of 64 participants per group. Given feasibility constraints, we enrolled 95 participants via convenience sampling, ensuring a minimum of 25 individuals per care setting. Exclusion criteria were (1) acute psychiatric emergency requiring involuntary hospitalization, (2) severe cognitive impairment (Mini-Mental State Examination score < 18) impeding questionnaire completion, (3) concurrent neurological disorders (e.g., stroke, dementia), and (4) inability or unwillingness to provide informed consent. Participants were stratified into “substance-using” or “non-using” cohorts based on self-report and corroborated by chart review.

### 2.3. Data Collection Instruments

Trained research assistants abstracted age, sex, marital status, employment, socioeconomic status (monthly income < USD 800), duration of psychiatric illness, primary DSM-5 diagnosis, and comorbid medical conditions from electronic medical records (EMR). A double-entry process with independent verification (discordance of < 2%) ensured data accuracy.

We adapted the World Health Organization’s ASSIST (Alcohol, Smoking, and Substance Involvement Screening Test) into Romanian, pilot-tested it on 10 individuals for clarity, and assessed internal consistency (Cronbach’s α = 0.82). The instrument quantified lifetime and past-three-month frequency, quantity, and duration of alcohol, marijuana, and inhalant use. “Inhalable substances” referred to volatile solvents (toluene-based glues, paint thinners) and nitrite “poppers”, consistent with WHO ASSIST categories. Substance-use status was coded dichotomously (yes/no) for primary analyses and categorized by substance type for subgroup comparisons.

SF-36 Questionnaire: We employed the Romanian-validated SF-36 v2.0 [21], which yields eight domain scores (physical functioning, role physical, bodily pain, general health, vitality, social functioning, role emotional, and mental health) and two summary measures (physical component summary and mental component summary). Scoring followed standard algorithms (0–100 scale), and instrument reliability in our sample was confirmed (α = 0.90).

Direct costs (inpatient days, outpatient visits, medication, and laboratory tests) were extracted from hospital billing systems and institutional accounting ledgers. Indirect out-of-pocket expenses (transportation, informal care) were captured via a short cost diary administered at study entry. All costs were recorded in Romanian leu (RON) and converted to USD using the average 2024 exchange rate (USD 1 = RON 4.60). We adjusted all costs for inflation to December 2024 values using the Romanian CPI.

### 2.4. Statistical Analysis

Analyses were performed in SPSS v28.0 (IBM Corp., Armonk, NY, USA). Continuous variables were tested for normality using the Shapiro–Wilk test and for homogeneity of variances via Levene’s test. Normally distributed data are presented as mean ± SD; non-normal data are reported as median (interquartile range). Categorical variables are reported as frequencies and percentages. Chi-square tests (with Yates’s correction where appropriate) compared proportions of substance use, gender, employment status, and diagnostic categories across groups. Independent-samples t-tests (or Mann–Whitney U tests for non-normal distributions) compared SF-36 scores and cost measures between substance-using and non-using participants. One-way ANOVA with Tukey’s post hoc tests (or Kruskal–Wallis with Dunn’s correction) evaluated differences among multiple substance-use subgroups. Concordance between self-reported substance use and chart documentation was substantial (Cohen’s κ = 0.81; 95% CI 0.70–0.92).

Pearson’s (or Spearman’s) correlation coefficients were used to assess relationships among substance use (binary), SF-36 total score, and total cost. We constructed a multiple linear regression model to identify independent predictors of overall SF-36 score, entering age, sex, illness duration, substance-use status, and care setting (inpatient vs. community/residential) simultaneously. Multicollinearity was screened using variance inflation factors; a VIF of >4 triggered removal. All variables retained in the final model exhibited VIF values of <2, indicating negligible collinearity. Statistical significance was set at two-sided *p* < 0.05. Effect sizes (Cohen’s d for t-tests; η^2^ for ANOVA) and 95% confidence intervals accompany key findings to contextualize clinical relevance.

## 3. Results

### 3.1. Demographics

Table 1 depicts the demographic characteristics of the 95 participants across three main care settings: inpatient psychiatric units, community-based centers, and residential facilities. The average age hovered around the early 40s, with a mean of 41.3 years overall. Notably, no significant difference emerged among the three settings regarding age (*p* = 0.352), suggesting a relatively comparable age distribution. Females accounted for nearly half of the sample (48.4%), with a similar proportion observed in each subgroup (46.9% inpatient, 50.0% community, and 48.0% residential).

Employment status varied significantly, however (*p* = 0.047). Only 25.0% of inpatients reported employment, compared to 42.1% in community settings and 44.0% in residential settings. This difference may reflect the constraints of hospitalization, which can disrupt regular work schedules and the ability to maintain stable employment. Interestingly, illness duration averaged 8.7 years overall, suggesting that most participants have been managing their psychiatric conditions for a considerable period, though the *p*-value of 0.621 indicates no meaningful differences among the three care environments. Marital status did not differ markedly, with roughly 41.1% reporting a stable partnership or marriage. Similarly, low-income status was reported by just over half of the participants (53.7%), a factor that may influence access to care and long-term treatment adherence. Given a mean illness duration approaching a decade, the cohort represents an entrenched clinical population whose longstanding functional impairment likely depresses baseline quality-of-life scores discussed below.

Table 2 illustrates the primary psychiatric diagnoses represented in the sample. Schizophrenia spectrum disorders constituted the largest share at 35.8%, suggesting a substantial proportion of participants with severe and chronic psychiatric conditions. Bipolar disorder followed at 24.2%, while major depressive disorder accounted for 20.0%. Schizoaffective disorder (9.5%) and other diagnoses, such as anxiety or post-traumatic stress disorder (10.5%), were also noted. When the chi-square test was used to compare diagnosis prevalence across the three care settings (inpatient, community, and residential), no statistically significant differences emerged (*p* = 0.266).

Table 3 categorizes substance-use patterns among the 95 participants. Over half (52.6%) reported no current or recent substance use, serving as a critical reference group for subsequent comparisons. Among those who used substances, alcohol was the most prevalent, with 19 individuals (20.0%) using only alcohol and an additional 9 (9.5%) reporting combined alcohol and marijuana use. Marijuana as a sole substance was documented by 7 individuals (7.4%), while inhalable substances were used by 10 participants (10.5%). Duration of use varied, ranging from an average of 3.8 years among those who used inhalable substances to 6.4 years in the alcohol-only group. These figures hint at the chronicity of substance involvement in certain cases, particularly long-standing alcohol use. Notably, the polysubstance category of alcohol plus marijuana (5.2 ± 2.6 years of use) indicates potential complexities in both treatment and relapse prevention.

### 3.2. Quality-of-Life Outcomes

Table 4 summarizes the SF-36 quality-of-life (QoL) outcomes for physical and mental-health dimensions, broken down by substance-use status. The no-use group reported the highest scores, with an overall SF-36 mean of 63.5. In contrast, participants who used alcohol and marijuana concurrently showed notably lower overall SF-36 scores (56.0), while those who used inhalable substances scored similarly low at 55.3. The ANOVA *p*-value of 0.027 suggests a statistically significant difference among these groups, indicating that substance use is indeed associated with reduced QoL.

Looking more closely, those who used alcohol only (58.2) or marijuana only (59.9) fell between the no-use group and the combined/polysubstance categories. Interestingly, the physical health dimension in alcohol-only users (58.4 ± 9.5) was somewhat lower than in marijuana-only users (60.9 ± 10.1), though the difference did not reach an independent significance threshold in post hoc tests. This suggests that while both groups exhibit reductions in QoL compared to non-users, the magnitude varies. Moreover, the mental-health component was particularly low in the alcohol + marijuana (55.1 ± 7.9) and inhalable (54.8 ± 8.7) groups, underscoring the potential psychological burden.

**Figure 1:**
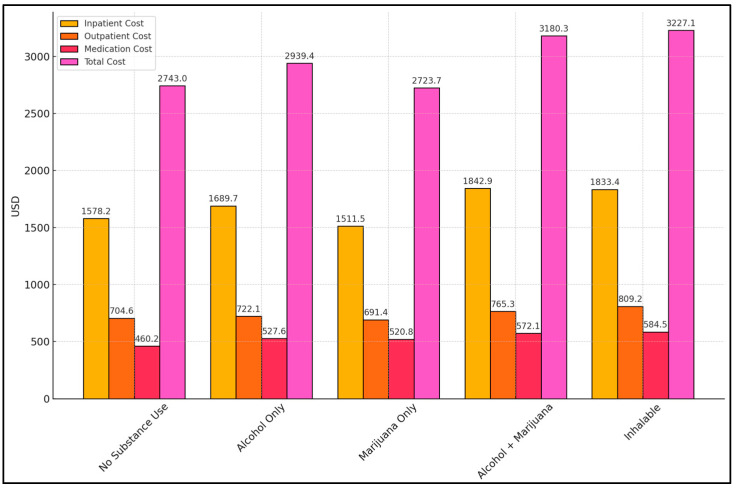
Cost analysis of psychiatric and substance-use care.

**Figure 2:**
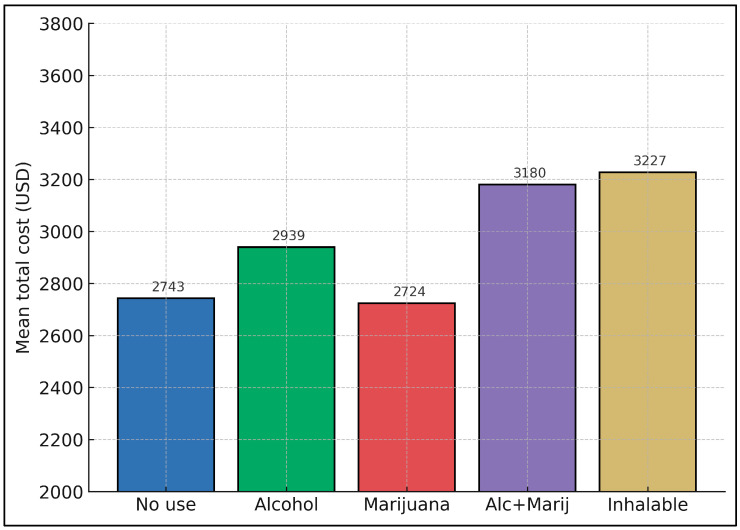
Total cost by substance use.

Figure 1 and Figure 2 detail the financial burden, converted from Romanian currency to USD, associated with psychiatric care across the various substance-use groups. Four cost categories were examined: inpatient hospitalization, outpatient visits, medication expenses, and overall total cost. Participants with no substance use had a total mean cost of USD 2743.0, which was significantly lower (*p* = 0.045) than the cost observed in the alcohol + marijuana (USD 3180.3) and inhalable (USD 3227.1) groups. Notably, the alcohol + marijuana and inhalable groups also incurred higher inpatient costs (USD 1842.9 and USD 1833.4, respectively) compared to the no-use group (USD 1578.2, *p* = 0.038 for inpatient cost).

While outpatient and medication costs did not differ significantly among groups (*p* = 0.311 and *p* = 0.112, respectively), there was a trend suggesting slightly elevated medication expenses in the combined substance-use cohorts. This could reflect the complex medication regimens needed to manage both psychiatric symptoms and potential withdrawal or relapse. Alcohol-only users (USD 2939.4 total) and marijuana-only users (USD 2723.7) sat between these extremes, indicating a moderate increase compared to non-users but not as high as the polysubstance or inhalable groups.

Table 5 compares clinical outcomes between participants without substance use (n = 50) and those reporting any form of substance use (n = 45). The readmission rate within a six-month window was substantially higher among the substance-using group (40.0% vs. 28.0%, *p* = 0.041). This aligns with the hypothesis that substance involvement exacerbates psychiatric instability, leading to increased hospitalization needs.

Mean length of stay also diverged significantly, with substance users averaging 15.3 days compared to 12.6 days for non-users (*p* = 0.023). This difference could reflect the additional complexity of managing withdrawal, medication interactions, or exacerbated psychiatric symptoms. A similar pattern emerged for relapse episodes, where 38.2% of substance users experienced at least one relapse in the preceding six months, compared to only 25.0% of non-users (*p* = 0.036). Emergency department (ED) visits served as another important indicator of acute psychiatric crises. Participants in the substance-using cohort averaged 1.8 ED visits (±0.7) over six months, notably higher than the 1.3 (±0.5) visits reported by non-users (*p* = 0.012).

Table 6 presents a Pearson correlation matrix capturing the interplay among substance use, quality of life (SF-36), and total costs. Substance use (coded as a binary variable: yes = 1, no = 0) correlated negatively with overall SF-36 scores (r = −0.28, *p* < 0.05), indicating that participants who reported using alcohol, marijuana, or inhalable substances tended to have a lower quality of life. This aligns with the earlier ANOVA findings in Table 4, reinforcing the notion that substance use may detract from both physical and mental well-being.

**Figure 3:**
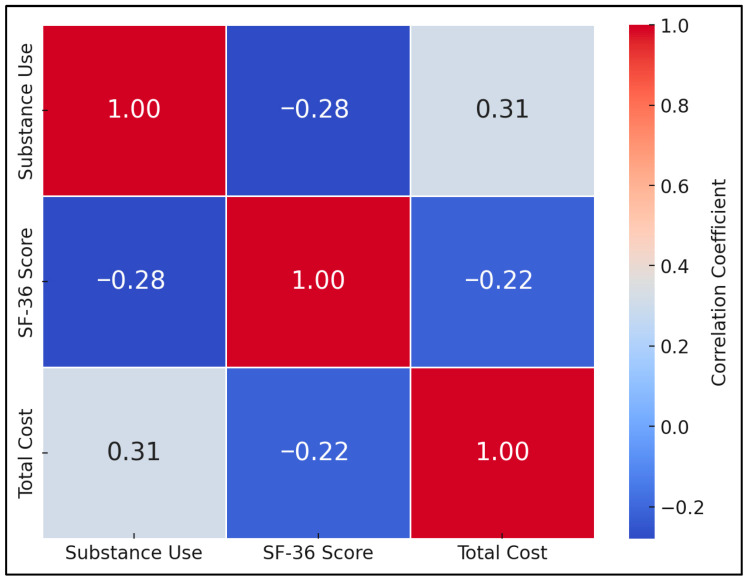
Correlation matrix.

A positive correlation emerged between substance use and total cost (r = +0.31, *p* < 0.05). This suggests that individuals reporting any form of substance use are associated with higher healthcare expenditures, likely driven by more frequent inpatient admissions, increased medication regimens, and possibly more complex psychosocial interventions. Interestingly, the overall SF-36 score showed a negative correlation with total cost (r = −0.22), though this particular value did not reach the threshold of statistical significance in our two-tailed test (Figure 3).

Table 7 showcases the results of a multiple linear regression model aimed at identifying factors that predict the overall SF-36 quality-of-life score among participants. The model includes age, sex, illness duration, substance-use status, and a dummy variable to indicate inpatient care versus other forms of care. Taken together, these variables explain approximately 21% of the variance in SF-36 scores (R^2^ = 0.21), and the model is statistically significant (F = 4.65, *p* = 0.001).

**Figure 4:**
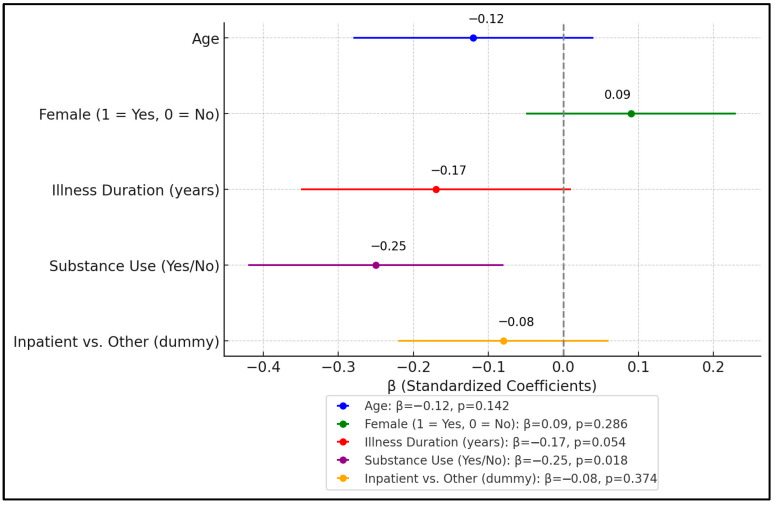
Forest plot analysis of β coefficients.

Notably, substance use emerged as a significant predictor (β = −0.25, *p* = 0.018), reinforcing earlier findings that substance use has a negative association with quality of life. Age and female sex showed only minor influences (β = −0.12, *p* = 0.142, and β = 0.09, *p* = 0.286, respectively), suggesting that demographic variables alone do not strongly dictate QoL in this sample. Illness duration approached but did not reach statistical significance (β = −0.17, *p* = 0.054), pointing to a possible trend that longer-standing psychiatric conditions could diminish quality of life. Interestingly, inpatient status compared to community or residential settings did not significantly predict SF-36 scores (β = −0.08, *p* = 0.374), implying that being hospitalized per se is not necessarily the key factor driving lower QoL (Figure 4).

A post hoc power analysis based on the observed group sizes (n = 45 vs. 50) yielded 79% power to detect the observed SF-36 difference (d = 0.50, α = 0.05). In sensitivity analyses, we repeated all tests with a binary exposure (any substance use versus none); the direction and significance of associations were unchanged (Table 8).

## 4. Discussion

This study offers an in-depth examination of psychiatric deinstitutionalization and highlights the role of substance use—alcohol, marijuana, and inhalable drugs—in influencing outcomes. We found that individuals reporting any substance use exhibited notably lower quality of life and higher healthcare costs than non-users. Moreover, substance use correlated with greater readmission rates, longer hospital stays, and elevated relapse frequencies, suggesting it acts as a potent modifier of clinical course. Deinstitutionalization, while beneficial for social reintegration, may inadvertently leave some patients vulnerable if community-based services are insufficient or fail to incorporate robust addiction interventions. Implementing these evidence-based interventions is feasible within Romania’s current staffing envelope. National workforce data indicate that one addiction counsellor typically covers three inpatient wards (≈150 beds). Embedding 15 min MI check-ins at discharge and a week 4 follow-up would require an additional 0.2 FTE per 50 community beds. The cost of upskilling ten nurses per ward via a 2-day CBT micro credential is ≈ EUR 150 per nurse—well within hospital continuing-education budgets.

These findings align with global trends showing that dual-diagnosis patients often require more intensive resources. Psychiatric disorders alone can be challenging to manage, yet co-occurring substance use imposes additional burdens on both the patient and the healthcare system. Several factors might contribute to this phenomenon: unrecognized withdrawal symptoms, inadequate adherence to psychiatric medications due to substance misuse, or complicated behavioral issues that impede stable community living. While some participants in community or residential settings reported higher employment rates and marginally better social function, the overall picture remained complex, particularly for those with polysubstance use.

Notably, these results underscored that substance use, more than demographic or some clinical variables, significantly predicted lower QoL scores. This highlights an opportunity for targeted interventions focusing on addiction treatment within psychiatric-care frameworks. Integrating motivational interviewing, cognitive-behavioral therapy for substance abuse, and close monitoring of relapse signs could be a valuable approach. In parallel, systematically implementing cost analyses can guide policymakers in resource allocation, emphasizing that front-loaded investment in specialized programs may prevent the recurrent expenses tied to repeated admissions and prolonged lengths of stay. Overall, addressing substance use is key to optimizing the potential benefits of deinstitutionalization efforts.

**Figure 5:**
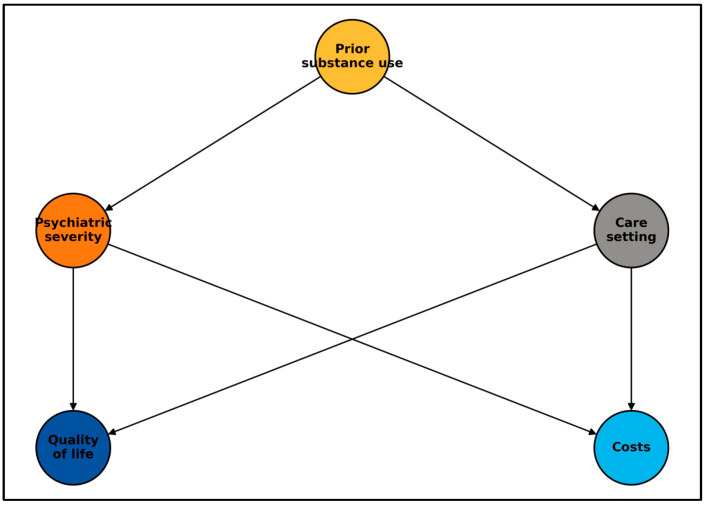
Directed acyclic graph (study causal framework).

To visualize the assumed causal structure, we constructed a directed acyclic graph (Figure 5). It positions prior substance use as a potential common cause of both (1) care-setting allocation and (2) psychiatric severity, which in turn influences quality of life and health-care costs. The DAG guided variable selection for multivariable models and underscores that our results indicate association, not causation.

The studies by Samartzis et al. and Fulone et al. contribute important insights into the process and challenges of psychiatric deinstitutionalization (PDI). Samartzis et al. conducted a scoping review that identified both barriers and facilitators affecting PDI efforts, such as inadequate planning and insufficient community-based alternatives as major obstacles, while effective facilitation involved strong advocacy and comprehensive community service [22,23]. Similarly, Fulone et al. utilized the SUPPORT tools to develop an evidence brief, which was instrumental in guiding policy dialogue aimed at enhancing care for deinstitutionalized individuals. Their study synthesized findings from 15 systematic reviews, highlighting strategies like psychoeducation and intensive case management that improved global status and reduced relapses and hospitalization [23]. Both studies emphasize the complexity of deinstitutionalizing psychiatric care and underscore the necessity for robust planning, stakeholder engagement, and sustained resources to successfully transition patients into community settings.

The studies by Gao and Olfson [24] and Peterson et al. [25] present compelling data on the economic burdens associated with mental-health care and substance-use disorders (SUD) in the United States, highlighting significant out-of-pocket (OOP) costs and hospital expenditures. Gao and Olfson [24] found that a significant portion of psychiatric outpatients, especially those below the federal poverty level, experience high OOP cost burdens despite federal policies intended to increase insurance coverage for mental-health care. Specifically, they reported that 2.4% of psychiatric outpatients had a high OOP burden, with this figure dramatically increasing to 12.8% among those below the poverty level. Patients with higher OOP costs were more likely to be uninsured or diagnosed with substance use or bipolar disorders. In a similar manner, Peterson et al. [25] conducted an economic evaluation revealing that the annual medical costs associated with SUD in U.S. emergency departments and inpatient settings exceeded USD 13 billion in 2017. Their analysis, based on over 158 million hospital encounters, identified specific costs by substance type, with alcohol-related disorders alone accounting for USD 7.6 billion.

When discussing the cost of healthcare, it is also important to assess the readmission risk among psychiatric patients with SUD. Nordeck et al. [26] focused on the rehospitalization patterns among patients who received consultation from a hospital-based SUD consultation-liaison team. Their findings indicated a significantly higher rate of rehospitalization among patients with opioid and cocaine use disorders compared to those without these diagnoses, with opioid use disorder alone showing a notably high adjusted odds ratio (AOR = 2.4) for rehospitalization. Similarly, Böckmann et al. [27] examined patient-level predictors of psychiatric readmission in a Swiss hospital, identifying several key factors such as previous admissions, comorbid psychiatric conditions like psychosis or mania, and higher clinical severity scores at discharge, which correlated with an increased likelihood of readmission within 12 months.

Similarly, Reif et al. [28] found that among the 30,439 Medicaid beneficiaries analyzed, two-thirds did not receive any follow-up services within 14 days of discharge. Those who did receive medication-assisted treatment (MAT) or residential treatment showed a statistically significant reduction in the risk of readmission within 90 days, with behavioral health admissions occurring in 29% of individuals with an index admission. This contrasts with the higher readmission risks linked to outpatient services and intensive outpatient services in specific models. On the other hand, Owusu et al. [29] reviewed 75 studies and found that interventions such as improved access to mental-health services, especially residential treatment, and effective crisis interventions substantially reduced readmission risks. This review highlighted that certain patient groups, especially those with learning disabilities, developmental delays, and substance abuse issues, were particularly prone to readmission, suggesting targeted interventions could significantly alter outcomes. Both studies underscore the importance of structured, accessible post-discharge care as a critical strategy to reduce healthcare costs and improve patient outcomes by directly addressing the factors that frequently lead to readmission.

A primary limitation of this study is its cross-sectional design, which restricts our ability to draw causal inferences about the relationships among psychiatric diagnoses, substance use, and outcomes. Longitudinal follow-up would better illuminate how substance-use patterns evolve and whether changes in use directly influence readmission rates and cost trajectories. Additionally, we relied on self-reported measures for substance use, which may be susceptible to social desirability bias or underreporting. Although we made efforts to verify participants’ psychiatric diagnoses and medications from medical records, inconsistencies in record-keeping could introduce classification errors. Our sample size of 95 participants, while sufficient for an exploratory analysis, limits the generalizability of the findings to wider national or global populations. Although our DAG informed regression adjusted for measured common causes, residual confounding cannot be excluded in cross-sectional data. Finally, the study took place in a single region of Romania, where local healthcare infrastructure and funding may differ substantially from other settings. Future research should encompass multiple sites and integrate robust longitudinal data collection.

## 5. Conclusions

The present study underscores the multidimensional complexity of psychiatric deinstitutionalization, particularly when co-occurring substance use is involved. While community-based care offers opportunities for greater autonomy and social engagement, these benefits can be overshadowed by the detrimental impact of alcohol, marijuana, and inhalable drugs. Our findings reveal that substance-using participants exhibit elevated healthcare costs, more frequent readmissions, and notably lower quality-of-life scores compared to their non-using counterparts.

Such outcomes highlight the necessity of integrating targeted substance abuse interventions within psychiatric care. By adopting a holistic treatment paradigm—encompassing psychosocial support, relapse prevention, and continuous monitoring—healthcare providers may mitigate the risk of relapse and reduce the economic burden on both patients and healthcare systems. Moreover, policymakers should be encouraged to fund specialized programs that directly address the interplay between mental illness and substance use, potentially leading to better adherence to psychiatric treatments and improved social reintegration. In essence, our data suggest that the success of deinstitutionalization efforts hinges on acknowledging and proactively managing substance use. Doing so can bridge the gap between policy intentions and real-world patient outcomes, creating a more effective and compassionate framework for long-term psychiatric care.

## Figures and Tables

**Table 1:** Demographic profile of participants.

Variable	Overall (n = 95)	Inpatient (n = 32)	Community (n = 38)	Residential (n = 25)	*p*-Value	Effect Size 95% CI
Mean Age (years)	41.3 (±11.2)	42.7 (±10.5)	39.6 (±11.9)	42.3 (±10.8)	0.352	η^2^ = 0.02 (0.00–0.08)
Female (%)	48.4	46.9	50	48	0.918	V = 0.03 (0.00–0.17)
Employed (%)	36.8	25	42.1	44	0.047	V = 0.23 (0.02–0.40)
Mean Illness Duration (years)	8.7 (±6.4)	9.5 (±6.3)	7.9 (±6.7)	8.8 (±6.2)	0.621	η^2^ = 0.01 (0.00–0.06)
Married/Partnered (%)	41.1	34.4	47.4	40	0.33	V = 0.11 (0.00–0.29)
Low Income (USD <800/month, %)	53.7	59.4	52.6	48	0.446	V = 0.14 (0.00–0.31)

η^2^ = partial eta-squared for one-way ANOVA, Cramer’s V for χ^2^ tests, n = 95, and post hoc power = 79% for primary QoL comparison.

**Table 2:** Distribution of psychiatric diagnoses.

Diagnosis	Overall (n = 95)	%
Schizophrenia Spectrum	34	35.8
Bipolar Disorder	23	24.2
Major Depressive Disorder	19	20
Schizoaffective Disorder	9	9.5
Other (e.g., Anxiety, PTSD)	10	10.5

Chi-square comparisons for each diagnosis across the three care settings were non-significant; *p* = 0.266 overall.

**Table 3:** Substance-use Patterns.

Substance Category	*n*	% of Total (n = 95)	Mean Duration of Use (Years, ±SD)
No Substance Use	50	52.6	–
Alcohol Only	19	20	6.4 (±2.9)
Marijuana Only	7	7.4	4.7 (±2.1)
Alcohol + Marijuana	9	9.5	5.2 (±2.6)
Inhalable Substances	10	10.5	3.8 (±1.7)

**Table 4:** Quality-of-life scores by substance-use status.

Group	SF-36 Physical (Mean ± SD)	SF-36 Mental (Mean ± SD)	Overall SF-36 (Mean)	*p*-Value (Overall)	Cohen’s d vs. No-Use (95% CI)
No Substance Use (n = 50)	62.7 (±10.3)	64.2 (±11.6)	63.5	–	reference
Alcohol Only (n = 19)	58.4 (±9.5)	57.9 (±9.7)	58.2	–	−0.50 (−1.03 to +0.04)
Marijuana Only (n = 7)	60.9 (±10.1)	58.8 (±12.2)	59.9	–	−0.33 (−1.12 to +0.47)
Alcohol + Marijuana (n = 9)	56.8 (±8.2)	55.1 (±7.9)	56	–	−0.71 (−1.43 to −0.01)
Inhalable (n = 10)	55.7 (±9.1)	54.8 (±8.7)	55.3	–	−0.77 (−1.46 to −0.08)
*p*-value (ANOVA)	–	–	–	0.027	

**Table 5:** Clinical outcomes and hospital readmissions.

Outcome	No Substance Use (n = 50)	Substance Use (n = 45)	*p*-Value
Readmission Rate (%) (6-month period)	28	40	0.041
Mean Length of Stay (days)	12.6 (±5.1)	15.3 (±6.4)	0.023
Relapse Episodes (% reporting ≥1)	25	38.2	0.036
ED Visits (past 6 months, mean)	1.3 (±0.5)	1.8 (±0.7)	0.012

**Table 6:** Correlation matrix: substance use, quality of life, and costs.

Variable	Substance Use	SF-36 Score	Total Cost
Substance Use (Yes/No)	–	r = −0.28 *	r = +0.31 *
SF-36 Score (Overall)	r = −0.28 *	−	r = −0.22
Total Cost (USD)	r = +0.31 *	r = −0.22	−

* Correlation is significant at *p* < 0.05 (two-tailed).

**Table 7:** Multiple linear regression predicting SF-36 score.

Predictor	β (Standardized)	*p*-Value	95% CI
Age	−0.12	0.142	−0.29 to +0.05
Female (1 = Yes, 0 = No)	0.09	0.286	−0.08 to +0.26
Illness Duration (Years)	−0.17	0.054	−0.34 to +0.01
Substance Use (Yes/No)	−0.25 *	0.018	−0.44 to −0.06
Inpatient vs. Other (Dummy)	−0.08	0.374	−0.25 to +0.09
Unstable Housing	−0.09	0.289	−0.27 to +0.09

* Statistically significant at *p* < 0.05, dependent variable: SF-36 overall score. Model statistics: R^2^ = 0.22, adjusted R^2^ = 0.17, F(6, 88) = 4.29, and *p* < 0.001.

**Table 8:** Binary exposure sensitivity.

Group	Cohen’s d	95% CI Lower	95% CI Upper
Alcohol	−0.50	−1.03	0.04
Marijuana	−0.33	−1.12	0.47
Alcohol + Marijuana	−0.71	−1.43	0.01
Inhalable	−0.77	−1.46	−0.08

## Data Availability

Data availability is subject to hospital approval.

## References

[1] Thornicroft G., Deb T., Henderson C. (2016). Community mental health care worldwide: Current status and further developments. World Psychiatry.

[2] Kohn R., Ali A.A., Puac-Polanco V., Figueroa C., López-Soto V., Morgan L., Barkil-Oteo A. (2018). Mental health in the Americas: An overview of the treatment gap. Rev. Panam. Salud Publica.

[3] Tyrer P. (2018). Has the closure of psychiatric beds gone too far? A view from the UK. BJPsych Bull..

[4] Fakhoury W., Priebe S. (2007). Deinstitutionalization and reinstitutionalization: Major changes in the provision of mental healthcare. Psychiatry.

[5] Lamb H.R., Bachrach L.L. (2001). Some perspectives on deinstitutionalization. Psychiatr. Serv..

[6] Regier D.A., Farmer M.E., Rae D.S., Locke B.Z., Keith S.J., Judd L.L., Goodwin F.K. (1990). Comorbidity of mental disorders with alcohol and other drug abuse: Results from the Epidemiologic Catchment Area Study. JAMA.

[7] Grant B.F., Stinson F.S., Dawson D.A., Chou S.P., Ruan W.J., Pickering R.P. (2004). Co-occurrence of 12-month alcohol and drug use disorders and personality disorders in the United States: Results from the National Epidemiologic Survey on Alcohol and Related Conditions. Arch. Gen. Psychiatry.

[8] Sacks S., Sacks J.Y., De Leon G., Bernhardt A.I., Staines G. (1997). Modified therapeutic community for mentally ill chemical “abusers”: Program description and preliminary findings. Subst. Use Misuse.

[9] Torrey E.F. (2015). Deinstitutionalization and the rise of violence. CNS Spectr..

[10] Mechanic D., Rochefort D.A. (1990). Deinstitutionalization: An appraisal of reform. Annu. Rev. Sociol..

[11] Anthony W.A., Cohen M.R., Farkas M.D., Gagné C. (2002). Psychiatric Rehabilitation.

[12] Buckley P.F. (2006). Prevalence and consequences of the dual diagnosis of substance abuse and severe mental illness. J. Clin. Psychiatry.

[13] Tew J., Ramon S., Slade M., Bird V., Melton J., Le Boutillier C. (2012). Social factors and recovery from mental health difficulties: A review of the evidence. Br. J. Soc. Work.

[14] Hunt G.E., Siegfried N., Morley K. (2019). Psychosocial interventions for people with both severe mental illness and substance misuse. Cochrane Database Syst. Rev..

[15] Drake R.E., Mueser K.T., Brunette M.F., McHugo G.J. (2004). A review of treatments for people with severe mental illnesses and co-occurring substance use disorders. Psychiatr. Rehabil. J..

[16] Colver A., Rapley T., Parr J.R., McConachie H., Dovey-Pearce G., Couteur A.L., McDonagh J.E., Bennett C., Maniatopoulos G., Pearce M.S. (2020). Facilitating transition of young people with long-term health conditions: Implications of a 5-year research programme. Clin. Med..

[17] Cechnicki A., Angermeyer M.C., Bielańska A. (2011). Anticipated and experienced stigma among people with schizophrenia. Soc. Psychiatry Psychiatr. Epidemiol..

[18] Tataru N. (2005). Psychiatry and geriatric psychiatry in Romania. Int. Psychiatry.

[19] Lam J.A., Rosenheck R. (1998). The effect of victimization on clinical outcomes of homeless persons with serious mental illness. Psychiatr. Serv..

[20] Druss B.G., Bradley K.A., Rosenheck R.A. (2000). Mental disorders and use of cardiovascular procedures after myocardial infarction. JAMA.

[21] Sima R.-M., Pleş L., Socea B., Sklavounos P., Negoi I., Stănescu A.-D., Iordache I.-I., Hamoud B.H., Radosa M.P., Juhasz-Boess I. (2021). Evaluation of the SF-36 questionnaire for assessment of quality of life in endometriosis patients: A systematic review and meta-analysis. Exp. Ther. Med..

[22] Samartzis L., Talias M.A. (2020). Assessing and improving quality in mental health services. Int. J. Environ. Res. Public Health.

[23] Fulone I., Barreto J.O.M., Barberato-Filho S., de Cássia Bergamaschi C., SilvaMarcus M.T., Lopes L.C. (2021). Improving care for deinstitutionalized people with mental disorders: Experiences using knowledge-translation tools. Front. Psychiatry.

[24] Gao Y.N., Olfson  M. (2025). High out-of-pocket cost burden of mental health care for adult outpatients in the United States. Psychiatr. Serv..

[25] Peterson C., Li M., Xu L., Mikosz C.A., Luo F. (2021). Assessment of annual cost of substance use disorder in US hospitals. JAMA Netw. Open.

[26] Nordeck C.D., Welsh C., Schwartz R.P., Mitchell S.G., Cohen A., O’Grady K.E., Gryczynski J. (2018). Rehospitalization and substance use disorder treatment entry among patients seen by a hospital SUD consultation-liaison service. Drug Alcohol Depend..

[27] Böckmann V., Lay B., Seifritz E., Kawohl W., Roser P., Habermeyer B. (2019). Patient-level predictors of psychiatric readmission in substance use disorders. Front. Psychiatry.

[28] Reif S., Acevedo A., Garnick D.W., Fullerton C.A. (2017). Reducing behavioural health inpatient readmissions for people with substance use disorders: Do follow-up services matter?. Psychiatr. Serv..

[29] Owusu E., Oluwasina F., Nkire N., Lawal M.A., Agyapong V.I.O. (2022). Readmission of patients to acute psychiatric hospitals: Influential factors and interventions. Healthcare.

